# Diagnostic Accuracy of Portal Vein Flow Velocity for Esophageal Varices in Cirrhotic Patients

**DOI:** 10.7759/cureus.43592

**Published:** 2023-08-16

**Authors:** Hafiz Muhammad Wasif Khan, Bushra Bilal, Kayenat Khan, Muhammad Osama Tariq Butt, Anas Ahmad Shah, Usman Iqbal Aujla

**Affiliations:** 1 Department of Gastroenterology and Hepatology, Pakistan Kidney and Liver Institute and Research Centre, Lahore, PAK; 2 Department of Radiology, Pakistan Kidney and Liver Institute and Research Centre, Lahore, PAK

**Keywords:** endoscopy, cirrhosis, portal vein flow velocity, doppler ultrasonography, esophageal varices

## Abstract

Background

Variceal bleeding is a life-threatening complication of cirrhosis. Traditionally, endoscopy has been utilized as a preferred modality for the detection and grading of esophageal varices. However, endoscopy is an invasive procedure and may not be readily available in resource-limited settings. To overcome this limitation, various non-invasive tests, including Doppler ultrasonography (DUS) with portal vein (PV) velocity measurement, have been investigated to predict the presence of esophageal varices (EV). This study aimed to evaluate the potential utility of portal vein flow velocity (PVFV) as a non-invasive alternative to endoscopic screening for predicting the presence of esophageal varices among cirrhotic patients.

Methodology

This validation cross-sectional study was carried out at the Department of Gastroenterology and Hepatology, Pakistan Kidney and Liver Institute & Research Centre (PKLI&RC), Lahore, Pakistan from June 8, 2022, to March 8, 2023. Cirrhotic patients were enrolled based on clinical, laboratory, and radiological assessments. Doppler ultrasonography was performed to measure portal vein flow velocity along other relevant indices. Subsequently, all patients underwent endoscopic evaluation to screen and grade the esophageal varices. Univariate and multivariate logistic regression analyses were performed to identify significant clinical predictors of EV based on the results of the independent sample t-tests or Mann-Whitney U tests. Receiver operating characteristic (ROC) curves were employed to determine the optimal cut-off value for portal vein flow velocity (PVFV). Sensitivity, specificity, positive predictive value (PPV), negative predictive value (NPV), and diagnostic accuracy were calculated based on the identified cut-off value. A p-value ≤ 0.05 was considered statistically significant.

Results

A cohort of 137 cirrhotic patients was enrolled. The study population consisted of 92 males (67.2%) and 45 females (32.8%). Endoscopic screening confirmed the presence of esophageal varices in 81 patients (59.91%). A multivariate analysis revealed that aspartate aminotransferase to platelet ratio index (APRI) (p=0.008) and portal vein flow velocity (p=0.001) were significant factors associated with esophageal varices and were used for receiver operating characteristic (ROC) analysis. The area under the curve (AUC) for PVFV was 0.981, and for APRI, it was 0.711. At a cut-off value of 18 cm/sec for PVFV, the sensitivity, specificity, positive predictive value (PPV), negative predictive value (NPV), and diagnostic accuracy for esophageal varices were found to be 93.83%, 92.86%, 95%, 91.23%, and 93.43%, respectively.

Conclusion

Measurement of portal vein flow velocity using Doppler ultrasonography (DUS) is a reliable screening method for predicting the presence of esophageal varices (EV) in patients with liver cirrhosis. DUS offers several advantages, including its non-invasive nature, cost-effectiveness, and widespread availability, making it a recommended approach due to its high diagnostic accuracy.

## Introduction

Cirrhosis of the liver is a chronic and progressive disorder which is characterized by the replacement of normal liver parenchyma by excessive fibrosis and scarring [1]. The etiology of cirrhosis is multi-factorial and includes non-alcoholic fatty liver disease (NAFLD), obesity, hepatitis B or C infection, excessive alcohol consumption, autoimmune disorders, cholestatic diseases, and an excess of iron or copper [2]. Liver cirrhosis poses a substantial burden on the healthcare systems across the globe and contributes significantly to the morbidity and mortality rates [3]. A formidable complication in the context of liver cirrhosis is the development of gastroesophageal varices, which carry the potential of life-threatening variceal haemorrhage [4]. Esophageal varices represent the most common type of gastroesophageal varices and are reported to be present in 50-60% of patients with compensated cirrhosis and their prevalence escalates up to 80% in patients with decompensated cirrhosis [5].

In patients with cirrhosis and portal hypertension the variceal bleeding accounts for 70% of upper gastrointestinal bleeding and stands as the second most common decompensating event after ascites [6]. The annual risk of variceal bleeding is estimated to range from 5-15% and the six-week mortality associated with each episode of variceal bleeding is up to 15-25% [6].

Management strategies in the presence of esophageal varices focus primarily on the prevention of variceal bleeding and treatment of acute variceal haemorrhage. The approach to the management of esophageal varices is individualized and is based on the degree of cirrhosis, grade of varices and patient’s specific clinical condition. Variceal screening has been recommended for individuals with cirrhosis who have clinically significant portal hypertension and in patients with decompensated liver disease. Endoscopic evaluation plays a pivotal role not only for screening the varices but also grading them for therapeutic guidance [7,8]. However, endoscopy is an invasive procedure [9], may not be readily available in resource-limited settings, and carries the risk of complications and may not be cost-effective for varices with low risk to bleed [4,10].

In patients with compensated cirrhosis, there is a growing emphasis on non-invasive methods to detect the gastroesophageal varices [10]. Various non-invasive markers are currently being studied to predict the existence of varices [9]. Non-invasive diagnostic techniques such as liver stiffness measurement, simple and multidetector computed tomography (CT), and magnetic resonance imaging (MRI) are being used for the diagnosis of esophageal varices and to predict the risk of bleeding in cirrhotic patients [11-13]. Doppler ultrasonography (DUS), which is a non-invasive method, has been investigated to establish a possible connection between portal hypertension (PH) and esophageal varices [14-16]. The current study aims to determine the role of portal vein flow velocity (PVFV) measurement by Doppler ultrasonography as a reliable and non-invasive screening method to triage cirrhotic patients requiring endoscopic assessment for their varices.

## Materials and methods

Study design and duration

This was a validation cross-sectional study conducted from June 8, 2022, to March 8, 2023 in Gastroenterology and Hepatology Department, Pakistan Kidney and Liver Institute (PKLI) & Research Centre, Lahore. This study was approved by the Institutional Review Board of Pakistan Kidney and Liver Institute (PKLI) & Research Centre, Lahore (PKLI-IRB/AP/72, dated 07-06-2022).

Inclusion criteria

Patients aged 18-80 years of either gender having cirrhosis of the liver due to various etiologies were enrolled in this study.

Exclusion criteria

Exclusion criteria for the study included patients with hepatocellular carcinoma, pre-existing portal vein thrombosis, previous endoscopic treatment for varices, and those currently receiving beta-blockers. Additionally, individuals with significant comorbidities such as end-stage renal disease, congestive heart failure, and severe respiratory or neurological ailments were also excluded from the study.

Sample size estimation and sampling technique

A study involving 137 patients was conducted, and the sample size was determined based on an estimated prevalence of 33.33% of esophageal varices (EV) [17]. The diagnostic accuracy of portal vein velocity (PVV) was considered to be 97% [17]. The sample size calculation used a margin of error of 5% and a confidence level of 95%.

Data collection procedure

Following approval from the Institutional Review Board (PKLI-IRB/AP/72), the study commenced after obtaining informed consent from the patients. The selection of patients was based on a comprehensive assessment that included clinical evaluation (medical history and physical examination), laboratory findings [serum bilirubin, aspartate aminotransferase (AST), alanine aminotransferase (ALT), serum albumin, alkaline phosphatase (ALP), gamma-glutamyl transferase (GGT), international normalized ratio (INR), complete blood count (CBC)], and radiological assessments (ultrasonography and computed tomography) to document presence of cirrhosis, portal vein diameter, portal vein flow velocity, presence of ascites, and spleen diameter.

All patients were evaluated and diagnosed as per the following protocol:

1. Initially, the presence of liver cirrhosis was established using the clinical, laboratory and radiological parameters.

2. After establishing the diagnosis of cirrhosis and fulfilling the selection criteria for this cohort, an abdominal Doppler ultrasound was performed to evaluate the velocity of portal vein flow along with other indices. Subsequently, all the patients were referred to the endoscopy unit for endoscopic screening and grading of esophageal varices (EV).

The operators performing the procedures were experienced consultants with a minimum of five years of expertise, and they were unaware of the patients’ clinical parameters, ensuring a blinded approach. To minimize variability between operators, the Doppler measurements of the portal system were conducted by a single operator. The measurements were performed in the morning before lunch to mitigate the potential impact of fluctuations in portal pressure throughout the day. The Siemens X 300 instrument was utilized to measure the portal vein flow velocity (PVFV) and portal vein diameter (PVD). During PVV measurements, the angle between the Doppler beam and the long axis of the portal vein was maintained below sixty degrees. The portal vein was scanned longitudinally while the patient was in the supine position, and the Doppler sample volume was positioned at the point where the portal vein intersected the hepatic artery. PVFV was recorded during a suspended expiration and averaged over a few seconds, with the sampling point adjusted to be in the center of the portal vein.

The patients underwent a standard endoscopic examination using an Olympus 190 videoscope from Japan. Prior to the procedure, the patients fasted for a minimum of 6 hours and underwent the examination in the morning before lunch. The esophageal varices (EV) were classified into three grades: Grade I if the varices were flattened by insufflation, Grade II if they could not be compressed with air insufflation and occupied less than one-third of the luminal diameter, and Grade III if they could not be compressed with air insufflation and occupied more than one-third of the luminal diameter.

All data collected was entered into IBM SPSS version 26 (IBM Corp., Armonk, NY, USA) for analysis. Quantitative data were expressed as mean ± standard deviation (if normally distributed) or median ± interquartile range (if not normally distributed). Categorical data were presented as frequencies and percentages. Independent sample t-tests (for normally distributed data) and Mann-Whitney U tests (for non-normally distributed data) were utilized to compare medians between patients with and without esophageal varices (EV). Univariate and multivariate logistic regression analyses were performed to identify significant clinical predictors of EV based on the results of the independent sample t-tests or Mann-Whitney U tests. Receiver operating characteristic (ROC) curves were employed to determine the optimal cut-off value for portal vein flow velocity (PVFV). Sensitivity, specificity, positive predictive value (PPV), negative predictive value (NPV), and diagnostic accuracy were calculated based on the identified cut-off value. A p-value ≤ 0.05 was considered statistically significant.

## Results

A total of 137 patients were enrolled in the study. Among them, 81 patients (59.91%) were diagnosed with EV, while 56 patients (40.9%) did not have EV. The distribution of EV grades was as follows: 30 patients (37.0%) had Grade I, 27 patients (33.3%) had Grade II, and 24 patients (29.6%) had Grade III esophageal varices. The mean age of patients in both the EV and non-EV groups was statistically similar (49.49 ± 14.025 years and 53.22 ± 9.40 years, p-value > 0.05). The study included 92 males (67.2%) and 45 females (32.8%). Among the EV cases, 60 (74.1%) were male and 21 (25.9%) were female, while among the non-EV cases, 32 (57.1%) were male and 24 (42.9%) were female, indicating a higher proportion of males in the EV group (p-value < 0.05) (Table 1). Among the EV cases, 42 (51.8%) cases had Child-Pugh class A, 28 (34.6%) had Child-Pugh class B and 11 (13.6%) had Child-Pugh class C. In the non-EV group, 37 (66.1%) had Child-Pugh class A, 15 (26.8%) had Child-Pugh class B and 4 (7.1%) had Child-Pugh class C. There was no statistically significant association between child Pugh class and esophageal varices, p-value > 0.05 (Table 1).

**Table 1:** Demographic and clinical profile of patients in relation to esophageal varices * Significant at 5% α; ** significant at 1% α a. Mann-Whitney U test, b. Independent sample t-test, c. Chi-square test ALT: Alanine transaminase; AST: Aspartate aminotransferase; ALP: Alkaline phosphatase; GGT: Gamma-glutamyl transferase; INR: International normalized ratio; ALBI: Albumin-Bilirubin Grade; GGT/Albumin: Gamma-glutamyl transferase/Albumin; Hb: Hemoglobin; PLT: Platelet Count; APRI: Aspartate aminotransferase to platelet ratio index; PVD: Portal vein diameter; PVFV: Portal vein flow velocity.

Demographic and clinical parameters			EV on Esophagogastroduodenoscopy		Test value	p-value
			Yes = 81 (59.1%)	No = 56 (40.9%)		
Age (years) (mean ± SD)			49.49 ± 14.025	53.22 ± 9.40	-0.633^b^	0.528
Gender; (f, %)		Male	60 (74.1%)	32 (57.1%)	4.30^c^	0.038*
		Female	21 (25.9%)	24 (42.9%)		
Child-Pugh class		A	42 (51.8%)	37 (66.1%)	3.053^c^	0.217
		B	28 (34.6%)	15 (26.8%)		
		C	11 (13.6%)	4 (7.1%)		
ALT (median ± IQR)			31.00 ± 32	30.0 ± 20	-0.837^ a^	0.403
AST (median ± IQR)			52.0 ± 59	36.0 ± 17	-3.955^ a^	<0.001**
ALP (median ± IQR)			158.0 ± 78	131.0 ± 99	-0.758^ a^	0.449
GGT (median ± IQR)			56.0 ± 74	90.0 ± 60	-1.075^ a^	0.282
Bilirubin (median ± IQR)			1.07 ± 1.54	0.70 ± 0.91	-2.964^ a^	0.003*
Albumin (median ± IQR)			3.28 ± 1.15	3.80 ± 1.0	-0.990^ a^	0.322
INR (median ± IQR)			1.28 ± 0.41	1.15 ± 0.32	-3.407^ a^	0.511
ALBI (mean ± SD)			2.04 ± 0.71	2.27 ± 0.71	-1.269^ b^	0.207
GGT/ Albumin (median ± IQR)			21.0 ± 18.90	23.0 ± 34.0	-0.657^ a^	0.511
Creatinine (median ± IQR)			0.70 ± 0.23	0.87 ± 0.59	-1.532^ a^	0.125
Sodium (median ± IQR)			1.37 ± 6.0	1.37 ± 6.0	-0.938^ a^	0.348
Hb (mean ± SD)			11.14 ± 2.04	11.95 ± 2.52	-0.853^ b^	0.395
PLT (median ± IQR)			1.07 ± 62.60	1.39 ± 145.0	-3.199^ a^	0.001**
APRI (median ± IQR)			1.30 ± 1.35	0.70 ± 0.90	-4.197^ a^	<0.001**
Spleen diameter (median ± IQR)			14.0 ± 3.0	12.0 ± 4.0	-5.089^ a^	<0.001**
PVD mm (median IQR)			12.0 ± 1.0	10.0 ± 1.20	-2.510^a^	0.012*
PVFV cm/sec (mean ± SD)			13.94 ± 2.61	20.96 ± 2.35	-14.896^ b^	<0.001**
Ascites; (f, %)	Yes		27 (33.8%)	11 (19.6%)	3.26^c^	0.071
	No		53 (66.2%)	45 (80.4%)		
Grades of EV	No		0 (0%)	56 (%)	137^ c^	<0.001**
	I		30 (37.0%)	0 (0%)		
	II		27 (33.3%)	0 (0%)		
	III		24 (29.6%)	0 (0%)		

The median levels of ALT, ALP, GGT, albumin, INR, GGT/albumin, creatinine, and sodium were statistically insignificant between the EV and non-EV groups (p-value > 0.05). The mean Albumin-Bilirubin grade (ALBI) and haemoglobin levels were also similar in both groups (p-value > 0.05). However, the median levels of AST, bilirubin, aspartate aminotransferase to platelet ratio index (APRI) and spleen diameter were significantly higher in the EV group compared to the non-EV group (p-value < 0.05). The median platelet count (PLT) was significantly lower in the EV group (p-value < 0.05). Similarly, the median portal vein flow velocity (PVFV) was significantly lower in the EV group compared to the non-EV group (p-value < 0.05) (Table 1).

**Figure 1:**
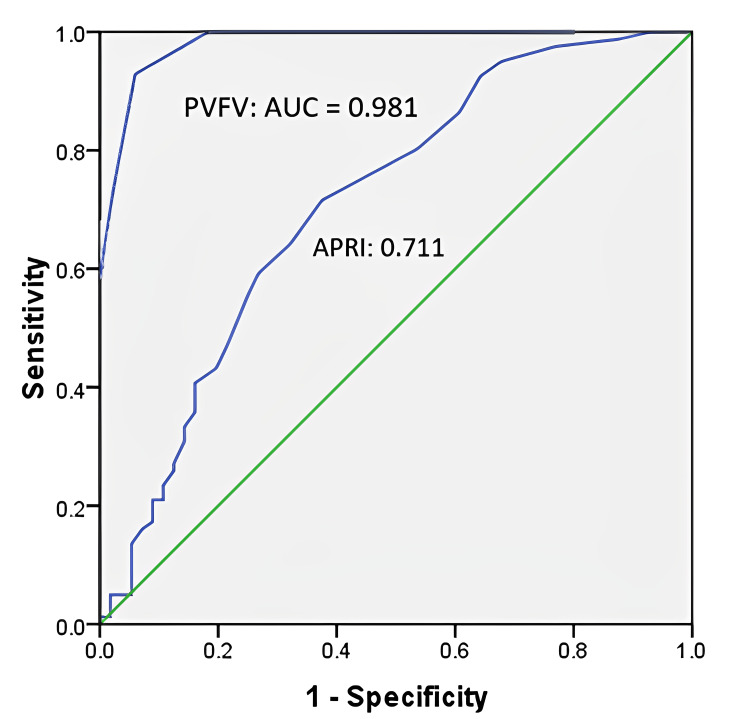
Receiver operative curve (ROC) showing area under the curve (AUC) for PVFV and APRI index PVFV: Portal vein flow velocity; APRI: Aspartate aminotransferase to platelet ratio index.

These significant clinical variables were used to screen for the possibility of EV using un-adjusted and adjusted odds ratio. Through multivariate analysis, only aspartate aminotransferase to platelet ratio index (APRI) and portal vein flow velocity (PVFV) were found to be significant and were used for the receiver operating characteristic (ROC) curve analysis (Table 2). The area under the curve (AUC) for PVFV was 0.981, and for APRI, it was 0.711 (Figure 1). The cut-off value for PVFV was determined as 18 cm/sec. At this cut-off value, the sensitivity, specificity, positive predictive value (PPV), negative predictive value (NPV), and diagnostic accuracy were calculated as 93.83%, 92.86%, 95%, 91.23%, and 93.43%, respectively (Table 3 and Figure 1).

**Table 2:** Univariate and multivariate logistic regression AST: Aspartate aminotransferase; INR: International normalized ratio; PLT: Platelet Count; APRI: Aspartate aminotransferase to platelet ratio index; PVD: Portal vein diameter; PVFV: Portal vein flow velocity.

Clinical parameters	β	p-value	Unadjusted (Univariate)	Adjusted (Multivariate)		
			OR	β	p-value	OR
AST	0.011	0.706	1.011 [0.956, 1.069]			
Bilirubin	0.056	0.859	1.058 [0.568, 1.971]			
INR	-0.593	0.695	0.552 [0.028, 10.720]			
PLT	0.001	0.933	1.001 [0.982, 1.020]			
APRI	1.022	0.153	2.779 [0.684, 11.290]	1.097	0.008	2.996 [1.338, 6.708]
Spleen	0.203	0.276	1.225 [0.850, 1.767]			
PVD	0.240	0.365	1.271 [0.756, 2.137]			
PVFV	-2.016	<0.001	0.133 [0.049, 0.362]	-2.116	<0.001	0.120 [0.045, 0.322]

**Table 3:** Diagnostic accuracy of PVFV to predict EV taking Esophagogastroduodenoscopy as gold standard Sensitivity = 93.83% (86.35, 97.33) Specificity = 92.86% (83.02, 97.19) Positive Predictive Value = 95% (87.84, 98.04) Negative Predictive Value = 91.23% (81.05, 96.19) Diagnostic Accuracy = 93.43% (87.99, 96.51) PVFV: Portal vein flow velocity; EV: Esophageal varices

		(EV) on Esophagogastroduodenoscopy			
	Cut-off value	Yes	No	Total	
PVFV	< 18	76	4	80	
	≥ 18	5	52	57	
Total		81	56	137	

## Discussion

Esophageal varices (EV) are a common complication of portal hypertension in patients with liver cirrhosis. Currently, upper gastrointestinal endoscopy is recommended for EV screening, but it is an invasive, costly, and expert-dependent procedure. To avoid unnecessary endoscopy, various non-invasive clinical, laboratory and radiological markers have been evaluated. Esophageal varices develop as a consequence of increased esophageal countercurrent from the portal vein, which is brought on by increasing intrahepatic vascular resistance and the amount of portal vein blood flow [18]. Abdominal Doppler ultrasound (DUS) has emerged as a promising non-invasive method to predict EV, as it allows visualization of portal pressure, an important factor in portal hypertension associated with liver cirrhosis. According to a research, DUS had overall good accuracy to predict EV i.e. sensitivity (88.98%), specificity (89.04%), positive predictive value (93.00%), negative predictive value (82.28%), and diagnostic accuracy (89.00%) [19]. In our study, the APRI index and portal vein flow velocity (PVFV) were identified as independent predictors of EV when applying multivariate and unadjusted odds ratios. Using receiver operating characteristic (ROC) analysis, it was determined that a cut-off value of 18 cm/s for PVFV yielded high sensitivity (93.83%), specificity (92.86%), positive predictive value (95%), negative predictive value (91.23%), and diagnostic accuracy (93.43%). A recent study reported that the cut-off value of PVV as ≥19 cm/s has 97% sensitivity [17]. Additionally, another study found that PVV had good diagnostic accuracy, with a sensitivity of 84% for detecting EV. Portal vein diameter (PVD) showed the highest specificity (55%) and negative predictive value (38%), along with a positive predictive value of 76% [4]. Collectively, these studies support the use of ultrasonography, particularly Doppler ultrasonography (DUS), as a reliable and non-invasive tool for identifying cirrhotic patients at risk of upper gastrointestinal variceal bleeding. By utilizing DUS, unnecessary invasive procedures can be avoided, and patients at high risk can be identified for further intervention or endoscopic evaluation.

We acknowledge some limitations in this study. Firstly, it was a single-observer, single-institution-based study. Multi-observer and multi-institution studies are further needed to establish the generalizability of our findings. Secondly, we only included patients with esophageal varices and the presence of portosystemic collaterals was not thoroughly evaluated. These collaterals may have an effect on velocity of portal vein blood flow. We encourage further studies in this regard.

## Conclusions

Portal vein flow velocity (PVFV) based on Doppler ultrasonography (DUS) is an effective screening method for predicting esophageal varices (EV) in patients with liver cirrhosis, with a cut-off value of 18 cm/s. DUS offers several advantages such as non-invasiveness, cost-effectiveness, and easy availability, making it a valuable tool with high diagnostic accuracy that should be utilized in clinical practice.
